# ICI-induced cardiovascular toxicity: mechanisms and immune reprogramming therapeutic strategies

**DOI:** 10.3389/fimmu.2025.1550400

**Published:** 2025-04-28

**Authors:** Jixuan Zheng, Yanyu Yi, Tingchen Tian, Shunming Luo, Xiao Liang, Yu Bai

**Affiliations:** Department of Reproductive Medicine, Key Laboratory of Birth Defects and Related Diseases of Women and Children, Ministry of Education, West China Second Hospital, West China School of Medicine, West China School of Pharmacy, Sichuan University, Chengdu, China

**Keywords:** immune checkpoint inhibitor, myocarditis, atherosclerosis, immune reprogram, metabolism, immune-related adverse effects

## Abstract

The advent of immune checkpoint inhibitors (ICIs) has revolutionized cancer treatment, offering life-saving benefits to tumor patients. However, the utilize of ICI agents is often accompanied by immune-related adverse events (irAEs), among which cardiovascular toxicities have attracted more and more attention. ICI induced cardiovascular toxicities predominantly present as acute myocarditis and chronic atherosclerosis, both of which are driven by excessive immune activation. Reprogramming of T cells and macrophages has been demonstrated as a pivotal factor in the pathogenesis of these complications. Therapeutic strategies targeting glycolysis, fatty acid oxidation, reactive oxygen species (ROS) production and some other key signaling have shown promise in mitigating immune hyperactivation and inflammation. In this review, we explored the intricate mechanisms underlying ICI-induced cardiovascular toxicities and highlighted the protective potential of immune reprogramming. We emphasize the roles of T cell and macrophage reprogramming in the heart and vasculature, showcasing their contributions to both short-term and long-term regulation of cardiovascular health. Ultimately, a deeper understanding of these processes will not only enhance the safety of ICIs but also pave the way for innovative strategies to manage immune-related toxicities in cancers therapy.

## 1 Introduction

The advent of ICIs has led to a radical transformation in the field of cancers treatment (1–3). By specifically inhibiting the inhibitory signaling pathways of T cells, ICIs unleash a powerful and robust immune response, demonstrating remarkable efficacy across a wide range of malignancies, from melanoma to lung cancer, thereby offering hope to many patients who previously faced limited options (1, 4, 5). However, despite their remarkable efficacy, the use of ICIs is also accompanied by a wide range of irAEs. With the continued increase of ICIs therapies, cardiovascular toxicity has emerged as an increasingly important clinical challenge (6–8).

IrAEs are expected complications of ICIs and can affect any system or organ in the body (7–9). ICIs induce these toxicities by blocking the checkpoints of immune self-tolerance, which leads to a cascade of inflammatory side effects. Although many of the inflammatory side effects are self-limiting and can be treated with hormonal shock therapy(short-term administration of high-dose corticosteroids which rapidly suppress inflammation mainly through inhibition of pro-inflammatory cytokine production and immune cell activation), there are still some less common yet potentially life-threatening toxicities, one of the most concerning being cardiovascular toxicity (10–12).

Although the cardiovascular toxicity is not the most frequent side effect of ICI therapy, it still poses a serious risk to patient health (7). Cardiovascular toxicity associated with ICIs can manifest as acute myocarditis, pericarditis, vasculitis, arrhythmias, etc., and long-term toxicity can cause chronic atherosclerosis (13–15). Besides, non-inflammatory cardiovascular toxicities including Takotsubo-like syndrome, asymptomatic non-inflammatory left-ventricular dysfunction, coronary vasospasm and myocardial infarction have been reported in individual cases (16).

Among these, ICI-induced acute myocarditis which is mainly resulted by the ICI-induced excessive T cell activation and pro-inflammatory cytokines secretion (17). Besides, ICI myocarditis is associated with the expansion of a specific population of gamma interferon (IFN-γ)-induced inflammatory macrophages (18). The ICI-induced acute myocarditis usually occurs shortly after ICI administration, with an incidence ranging from 0.27% to 2.46%. The incidence is higher in patients receiving combination ICI therapy (1.3%), and the mortality rate associated with this condition can reach as high as 30-50% (17, 19, 20). Furthermore, long-term use of ICIs has been found to be associated with atherosclerosis. Early clinical data support this concern: data from a matched cohort study indicate a threefold higher incidence of atherosclerotic cardiovascular events in the 2 years following ICI therapy compared with a similar pretreatment time frame. Imaging also shows that the rate of progression of total aortic plaque volume was > 3-fold higher with ICIs (from 2.1%/year pre to 6.7%/year post) (21). The ICI-induced atherosclerosis is mainly driven by pro-inflammation macrophage polarization, abnormal T cell differentiation and oxidative stress (13, 22, 23).

In summary, ICI-induced myocarditis and atherosclerosis are mediated by irregular immune reaction. Both conditions involve uncontrolled activation of immune cells, which leads to inflammation, tissue damage and long-term cardiovascular dysfunction (24–26). In clinical practice, with increased ICI therapy applied to a broader range of cancers, ICI-induced cardiovascular toxicity has garnered growing attention. Therefore, the effective management of both acute and chronic cardiovascular events has become a major clinical challenge. There is an urgent need to enhance our understanding of the mechanisms underlying ICI-induced cardiovascular toxicity. This knowledge is crucial to develop new therapeutic strategies that can effectively manage cardiovascular toxicity while preserving the anticancer efficacy of ICIs in clinical applications.

Immune reprogramming refers to the process modulating immune cell function and phenotype to support specific immune functions and adapt to the microenvironment (27). Immune reprogramming therapy has emerged as a promising strategy in cancer therapy, offering the potential to modulate the tumor microenvironment and enhance the anti-tumor immune response. By reprogramming immune cells, it is feasible to surmount immune evasion strategies utilized by tumors, thereby enhancing the effectiveness of immunotherapy (28). Furthermore, it could serve a vital function in mitigating the immunotoxicity frequently linked to immunotherapy, thus reducing adverse effects by immunoregulation and elevating the overall quality of life for individuals receiving such therapies (29).

In the case of ICI-induced cardiovascular toxicity, immune reprogramming can provide protection through several mechanisms. Firstly, it can enhance the flexibility of immune cells, allowing for improved regulation of inflammatory responses that contribute to cardiovascular damage (30). By shifting the state of immune cells towards anti-inflammatory pathways, immune reprogramming can mitigate excessive immune activation and tissue injury (31). Secondly, this reprogramming can bolster the survival and function of cardioprotective immune populations, such as regulatory T cells (Tregs), which play a pivotal role in maintaining cardiac homeostasis (32). By promoting a favorable environment for these cells, immune reprogramming can help counteract the detrimental effects of ICIs on cardiac tissue, thereby reducing the risk of toxicity and improving overall cardiovascular health during cancer immunotherapy.

In this review, we summarized the potential mechanisms of ICI induced cardiovascular toxicity and presented a diverse range of possible immune reprogramming strategies to mitigate the risks associated with cardiovascular events. Therefore, we mean to provide theoretical support for further research endeavors that seek to enhance the clinical application and safety of ICIs, ultimately contributing to better patient outcomes.

## 2 Cardiovascular toxicity - mechanism and traditional treatment

### 2.1 Short-term cardiovascular toxicity: ICI-induced acute myocarditis

Clinically, myocarditis can arise from a diverse array of infectious agents, such as viruses and bacteria, as well as non-infectious causes, including autoimmune diseases and certain medications (33). ICI-induced acute myocarditis can present with a wide range of symptoms, varying from asymptomatic elevation in cardiac biomarkers to end-organ failure. Clinical manifestations may include chest pain, dyspnea, myalgia, myasthenia, ptosis, muscle weakness, syncope, palpitations, pulmonary edema, and even cardiogenic shock (34–36).

As precise etiology of ICI-myocarditis remains uncertain, the current therapeutic strategies are mainly relied on non-specific immunosuppression, primarily corticosteroids. However, these agents have significant side effects including osteoporosis and for Pneumocystis jirovecii pneumonia and are often inadequate for severe cases (37–40). The use of corticosteroids may broadly suppress immune function, thereby diminishing the antitumor efficacy of ICIs (37, 39, 41). Moreover, despite the timely use of high-dose corticosteroids, 67% patients (16 out of 24) still developed corticosteroid resistance (37). Consequently, to effectively treat ICI myocarditis, it is of utmost importance that we urgently deepen and expand our understanding of its complex pathogenesis.

Current researches have suggest that viral infections can lead to myocardial injury through mechanisms such as apoptosis and necrosis of cardiomyocytes by disrupting critical cellular functions with (42). What’s more important, the pathogenesis of viral myocarditis may also involve aberrantimmune responses against cardiac autoantigens, indicating a possible autoimmune dysregulation (43, 44). As a result, though the precise etiology of ICI-myocarditis remains uncertain, the parallels drawn from conventional myocarditis mechanisms could help elucidate the underlying pathogenesis, highlighting the potential for immune dysregulation and autoimmune responses as critical factors in the development of this adverse effect associated with cancer immunotherapy.

Recent studies have elucidated the clinical manifestations of ICI-myocarditis and revealed its pathogenesis through studies in animal models, especially the important role of immune checkpoint molecules in cardiac antigen tolerance. In conjunction with preclinical models, current evidence supports a potential model in which self-reactive cardiac T cells may arise due to a lack of specific cardiac antigen expression in thymic epithelial cells that disrupts central tolerance (45).

Normally, T cells require antigen recognition via the T cell receptor (TCR) and co-stimulatory signals generated by the binding of CD28 on T cells to CD80/CD86 on antigen-presenting cells (APCs) (46). ICIs work by blocking immune inhibitory molecules such as CTLA-4 and PD-1, thereby relieving the suppression on T cells and allowing for enhanced activation. This inhibition relief enables T cells to receive stronger co-stimulatory signals (such as CD28 binding to CD80/CD86) within lymphoid tissues, promoting the activation and proliferation of self-reactive T cells (46, 47). Once activated, these T cells circulate to peripheral tissues, where they recognize specific antigens, such as cardiac antigens, via TCR- major histocompatibility complex(MHC) interactions, and exert effector functions. This process triggers clonal expansion of T cells specifically targeting cardiac antigens, leading to myocardial injury (46, 47). Physiologically, peripheral tolerance, which is maintained by immune checkpoints, would effectively inhibit the activation of these potentially self-reactive T cells. However, the introduction and use of ICIs disrupt this crucial immunomodulatory regulatory mechanism, leading to an increased risk of autoimmunity and the activation of these T cells (18, 48, 49). Furthermore, dysfunction of immunomodulatory cells contributes to the uncontrolled proliferation of autoimmune cells. In ICI-induced myocarditis, the loss of Treg function results in uncontrolled expansion of CD8+ T cells, further exacerbating autoimmune myocarditis (50).

In addition to T cells, macrophages also play a crucial role in ICI-induced myocarditis. Wei et al. suggested that premature death in robust preclinical ICI-induced myocarditis mouse model is associated with myocardial infiltration by both T cells and macrophages (51).Activation of T cells and activation of pathways such as janus kinase (JAK)/signal transducer and activator of transcription (STAT) and nuclear factor kappa B (NF-κB), as well as the secretion of cytokines (particularly IFN-γ) subsequently stimulate C-X-C motif chemokine ligand 9+(CXCL9+)CXCL10+ macrophages (18). These macrophage-secreted chemokines act as chemoattractants for CXCR3-expressing effector T cells, prompting them to infiltrate cardiac tissue and reinforcing positive inflammatory feedback (18). Besides, studies have shown that reprogramming macrophages from the proinflammatory M1 phenotype to the anti-inflammatory M2 phenotype can significantly reduce myocardial inflammation. Mechanically, the PD-1 inhibitor exerted its effect in promoting M1 polarization and cardiac injury by modulating the miR-34a/KLF4-signaling pathway. Furthermore, the reversed M1 polarization showed good potential to improve cardiac injury *in vivo (* 52, 53). Therefore, this shift, which relies on polarization reprogramming, is a promising therapeutic strategy that can reduce immune-mediated damage while maintaining cardiac function.

**Figure 1:**
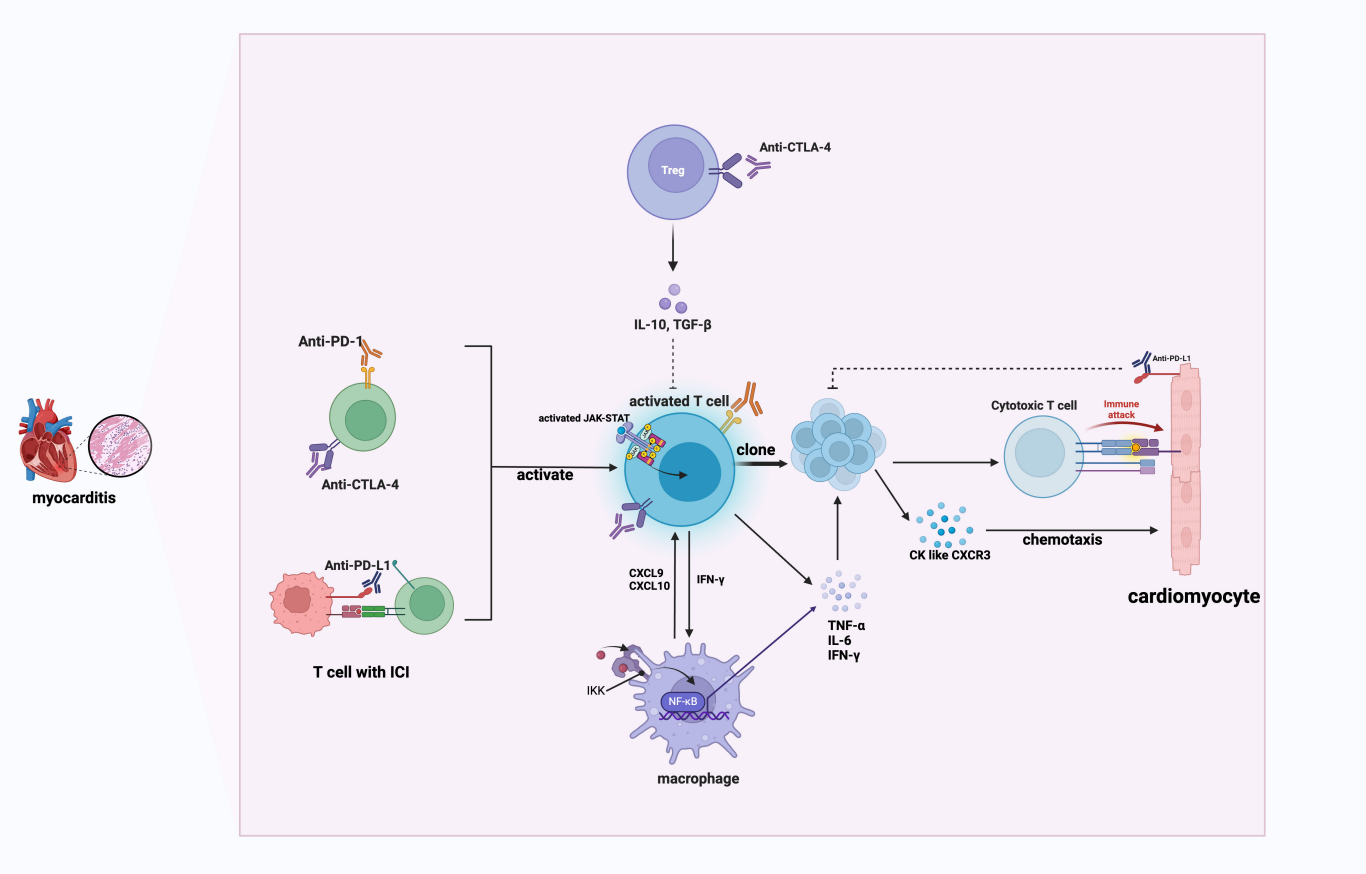
The mechanism of ICI-induced myocarditis. This schematic illustrates immune checkpoint inhibitor (ICI)-mediated myocarditis involving activated T cells, macrophages, and cytokine-mediated inflammatory responses. PD-1, programmed cell death protein 1; CTLA-4, cytotoxic t lymphocyte-associated antigen-4; IL, interleukin; TGF-β, Transforming Growth Factor Beta; JAK, Janus Kinases; STAT, Signal Transducer and Activator of Transcription; IKK, inhibitor of κB kinase; CXCL9, C-X-C motif chemokine ligand 9; CXCL10, C-X-C motif chemokine ligand 10; IFN-γ, Interferon gamma; IFN-γ, Interferon gamma; CK, chemokines; CXCR3, C-X-C motif chemokine receptor 3. Activated T cells produce IFN-γ, stimulating macrophages through the JAK/STAT pathway. Activated macrophages secrete CXCL9 and CXCL10, attracting CXCR3-expressing effector T cells to cardiac tissues, creating a positive inflammatory feedback loop that exacerbates cardiac inflammation.

In conclusion, T cell, macrophages and the inflammatory cytokines they secreted work together to mediate ICI-induced myocarditis, as illustrated in **Figure 1**. As a result, T cell and macrophage reprogramming offer promising strategies for the treatment of refractory myocarditis. Further research into immune modulation and the development of precision medicine approaches have the potential to improve outcomes for patients with ICI-related immune-mediated cardiovascular toxicity.

### 2.2 Long-term cardiovascular toxicity: atherosclerosis induced by ICI

With the extensive long-term use of ICI agent, its long-term cardiovascular toxicities especially atherosclerosis has also been observed. Atherosclerosis is now recognized as a chronic inflammatory disease, the immune response plays an important role in the formation and progression of plaques (54–56). Recent clinical data suggest that the use of ICIs is associated with accelerated atherosclerosis and atherosclerotic cardiovascular events, including myocardial infarction and stroke (21). Some case reports have also linked PD-L1 and PD-1 inhibitors to the rapid progression of coronary heart disease and fatal acute coronary syndrome due to lung malignancies and giant cell tumors of bone (57, 58). In addition, some small-scale human imaging and histological studies have attempted to confirm that ICI treatment may increase atherosclerosis inflammation and accelerate the formation of atherosclerotic plaques (21, 59, 60).

**Figure 2:**
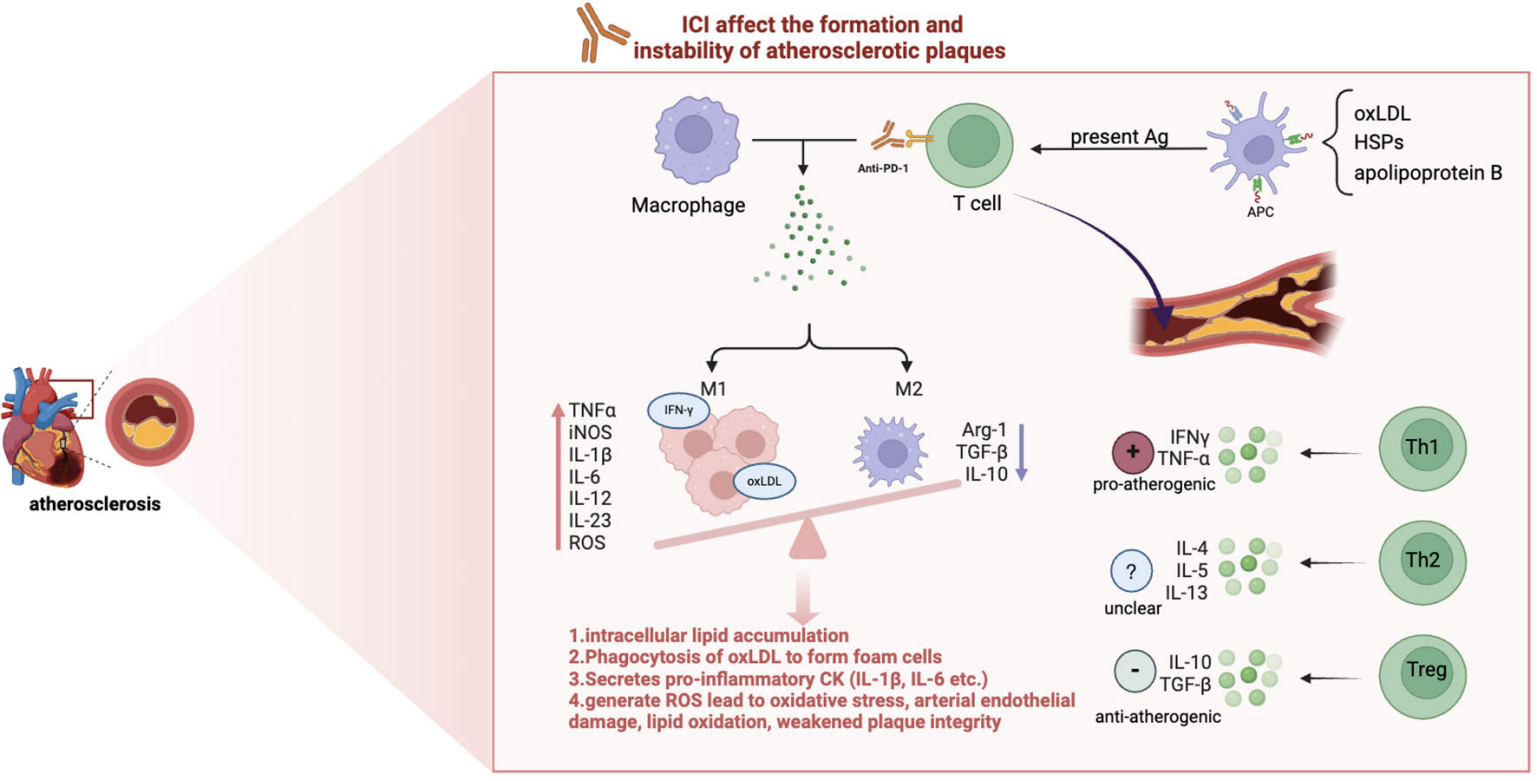
The mechanism of ICI-induced atherosclerosis. Immune checkpoint inhibitors (ICIs) enhance vascular inflammation by influencing T cell and macrophage. oxLDL, Oxidized Low-Density Lipoprotein; HSPs, Heat Shock Proteins; Ag, Antigen; APC, Antigen-Presenting Cells; Th, T Helper cells; Arg-1, arginase-1; TNFα, Tumor Necrosis Factor alpha; iNOS, Inducible Nitric Oxide Synthase; IL, Interleukin; ROS, Reactive Oxygen Species(highly reactive molecules that contribute to vascular inflammation and tissue injury);IFN-γ, Interferon gamma. M1 macrophages, activated by pathways involving NF-κB and JAK/STAT, secrete pro-inflammatory cytokines (e.g., IL-1β, IL-6, TNF-α) and reactive oxygen species (ROS), promoting plaque instability and progression. In contrast, M2 macrophages mediate anti-inflammatory responses, favoring plaque stability and regression.

The connection between ICI and atherosclerosis has been examined in several studies (21, 61, 62). In brief, while suppressing cancer, ICI agents may lead to an enhanced inflammatory response within atherosclerotic plaques by relieving the inhibitory effect on T cells and reprogramming macrophages towards pro-inflammation phenotype, resulting in increased plaque instability and ultimately an increased incidence of cardiovascular events such as myocardial infarction and stroke (24, 25, 61), as illustrated in **Figure 2**.

Macrophages are plastic and can be influenced by the local cytokine environment, resulting in the differentiation of these cells into an inflammatory (M1) or anti-inflammatory (M2) phenotype (63). The balance between M1 and M2 macrophages is crucial in determining the overall outcome of atherosclerotic disease. However, ICI treatment has the effect of tilting the balance in favor of the M1 phenotype. Macrophages of the M1 phenotype, which are influenced by factors such as free fatty acids, oxidized lipids and IFN-γ, are the predominant type observed in early atherosclerotic lesions (54). These macrophages are responsible for intracellular lipid accumulation, foam cell formation and the secretion of pro-inflammatory cytokines, including interleukin-1β (IL-1β) and interleukin-6 (IL-6) (64, 65). It is noteworthy that when M1 macrophages phagocytose oxidized low-density lipoproteins (oxLDL), they undergo conversion into foam cells, which represents a pivotal factor in plaque instability. In contrast, M2 macrophages are induced by cytokines such as interleukin-4 (IL-4), interleukin-13 (IL-13) and interleukin-10 (IL-10), which promote collagen formation and effective removal of lipids, thereby promoting plaque regression (66). Therefore, therapeutic strategies targeting macrophage subset polarization may hold promise for not only preventing atherosclerosis progression but also for promoting regression of established plaques.

The presence of various T cell subsets in atherosclerotic plaques, such as CD8^+^ cytotoxic T cells, T helper 1 (Th1) cells, Th2 cells, Th17 cells and Treg cells, indicates that the immune system plays a complex role in the development of atherosclerosis (67). APCs present atherosclerosis-associated antigens, including oxLDL, heat shock proteins and apolipoprotein B, to naïve T cells in lymphoid tissue (23, 67, 68). This antigen presentation is crucial for driving the differentiation of naive T cells into effector T cell subsets, particularly CD4+ T helper (Th) cells and CD8+ cytotoxic T cells.

Activated CD4+ T cells can further produce pro-inflammatory cytokines like IFN-γ and IL-17. These cytokines can promote the activation of macrophages and lead to increased foam cell formation and plaque instability (69–71). ROS generated by foam macrophages can lead to oxidative stress, damaging the arterial endothelium, promoting lipid oxidation, and weakening plaque integrity (72, 73). Additionally, pro-inflammatory cytokines such as IFN-γ and IL-6 promote smooth muscle cell apoptosis and stimulate the secretion of matrix metalloproteinases (MMPs) by T cells and macrophages (74). These MMPs degrade the extracellular matrix in the arterial wall, further destabilizing plaques and increasing the risk of plaque rupture, leading to cardiovascular events such as myocardial infarction and stroke (75).

CD8+ T cells, on the other hand, directly contribute to tissue damage and lesion instability through cytotoxic mechanisms, such as the release of perforin and granzymes, which can induce apoptosis in vascular smooth muscle cells and other cells within the plaque (76). This cytotoxic activity can further destabilize plaques, making them more prone to rupture and potentially leading to acute cardiovascular events like myocardial infarction or stroke.

In summary, the heightened activation of T cells may lead to increased recognition of atherosclerosis-associated antigens, resulting in enhanced inflammatory responses within plaques and cardiovascular complications in patients undergoing ICI treatment. Consequently, monitoring T cell activity and the resulting inflammatory response becomes critical in patients receiving ICI therapy.

From a therapeutic perspective, with excellent performance in lowering low-density lipoprotein (LDL) and anti-inflammatory, statins have played a key role in the prevention and treatment of atherosclerosis (77). In addition, there are several combination treatment options that are currently receiving significant attention for their potential to enhance the antihypertensive effect of statins. For instance, proprotein convertase subtilisin/kexin type 9(PCSK9) mutation have been identified as the cause of autosomal dominant familial hypercholesterolemia, PCSK9-targeted inhibitors significantly reduce the level of this highly atherogenic lipoprotein in the blood by promoting the recycling of undegraded LDL receptors to the cell surface, which further captures and removes LDL (78, 79). These inhibitors can further improve the cardiovascular prognosis of patients already treated with statins. In addition, drugs that target upstream of the mechanism of action of statins, such as inhibitors of hydroxymethylglutaryl-coenzyme A (HMG-CoA) reductase (such as bezafibrate), have also been approved, making the choice of non-statins lipid-lowering drugs more diverse (80).

However, in clinical practice, the therapeutic efficacy of traditional agents is limited. This may be due to their rapid clearance and unsatisfactory accumulation at the arterial injury site (81). Given the important role of immune cells in atherosclerosis, anti-inflammatory therapies also show potential in reducing cardiovascular events. The Canakinumab Anti-inflammatory Thrombosis Outcome Study (CANTOS) study showed that the risk of recurrent cardiovascular events can be effectively reduced in patients with a history of atherosclerosis by using interleukin-1β inhibitors such as canakinumab (82).In addition, antiplatelet agents (e.g. aspirin) are widely used to prevent cardiovascular events by stabilizing atherosclerotic plaques, especially ‘vulnerable plaques’ that are prone to rupture (83).

In summary, the current treatment plans for ICI induced cardiovascular toxicity often focuses on symptomatic treatment, aimed at relieving symptoms and managing acute events. Although these regimens are effective in acute management and the long-term application may cause side effects such as abnormal glucose metabolism and osteoporosis (41). These methods cannot completely control the chronic immune response caused by ICI, and fail to fundamentally regulate the overactivity of the immune system (84). Thus, regulating the function of immune cells, such as T and macrophages, is an important therapeutic direction. Future therapeutic approaches may include strategies to modulate immune cell responses within atherosclerotic lesions, thereby providing a dual benefit of reducing cardiovascular risk while maintaining effective cancer treatment protocols.

## 3 Immune reprogramming

Immune remodeling is a prominent feature of cardiovascular disease and neoplasia (85). Immune reprogramming therapies which regulate the immune response through cellular pathways, play a crucial role in ICI-induced cardiovascular toxicity therapies and ultimately result in a significantly lower overall impact on the patient’s immune system than traditional immunosuppressive therapies (85, 86). By regulating specific immune pathways, the risk of cardiovascular toxicity can be reduced while maintaining the anti-tumor effect of ICI, making it more sui for long-term use in the management of cardiovascular toxicity in cancer patients. Specifically, targeting key pathways in T cells and macrophages can effectively reduce inflammation and tissue damage caused by excessive immune responses (85).

### 3.1 T cell reprogramming therapy for ICI-induced cardiovascular events

#### 3.1.1 Immune reprogramming targeting glycolysis in T cell

Metabolic reprogramming plays an important role in the activation, proliferation, differentiation and migration of immune cells, and profoundly affects the progression of heart disease. Glycolysis is a conserved and strictly regulated biological metabolic process that provides essential energy and metabolic intermediates to the body by breaking down glucose into pyruvate (87). After the T cell receptor is stimulated, the initial T cells undergo metabolic remodeling through glycolysis, thereby effectively differentiating into effector cell populations (88). Specifically, the differentiation of Th1 and Th17 cells depends on glycolysis to meet the high energy and biosynthetic requirements, while Tregs prefer oxidative phosphorylation and fatty acid oxidation to maintain their function (89). The glycolytic pathway plays a key role in the differentiation, proliferation and function of Th17 cells. Studies have shown that blocking glycolysis in mice by drugs or genetic knockout can lead to a lack of transcriptional signals in Th17 cells, thereby preventing the development of autoimmune diseases (90). Therefore, immunometabolic reprogramming targeting glycolysis may effectively regulate the metabolic homeostasis of T cells, providing a new therapeutic strategy for intervening in the cardiovascular toxicity caused by ICI and maintaining anti-cancer efficacy.

Naïve T cells primarily rely on oxidative phosphorylation (OXPHOS) for energy production (91). However, after activated by antigens, metabolic reprogramming occurs in naïve T cells and support them differentiate into effector T cells. This metabolic shift results in a reliance of T cells on aerobic glycolysis to fulfill the heightened energy needs required for rapid cell proliferation and effector functions (92–94). In ICI-induced myocarditis, targeting the glycolytic pathway in these T cells may help mitigate their proinflammatory activity. Axelrod et al. indicated the key role of CD8 T cells in the pathophysiology of the disease by depleting CD8^+^ T cells in mice to improve survival benefits (95). However, inhibiting glycolysis in CD8^+^ T cells can impair their anti-tumor function which is rely on glycolysis too (96). Recent studies have demonstrated that metabolic reprogramming of CD8^+^ T cells through glycolysis inhibition, such as by deleting pyruvate kinase muscle 2 (PKM2), can shift these cells toward a TCF1^+^ progenitor-like state, enhancing their persistence and responsiveness to PD-1 blockade therapy. While this metabolic shift may improve the durability of the anti-tumor immune response, it could also dampen the immediate effector functions of CD8^+^ T cells, which are crucial for tumor elimination (97). In addition, glycolysis inhibitors such as 2-deoxy-D-glucose (2-DG), 3PO, and PFK158 have shown promise in reducing immune-mediated damage by dampening excessive glycolytic activity in T cells. Nonetheless, they need to be used with caution to avoid compromising antitumor immunity (98–101). Therefore, whether the inflammatory effect of ICI on the heart muscle can be reduced by inhibiting CD8^+^ T cells, namely how to keep the balance between tumor-killing ability and cardiovascular protection should be the focus of our future research.

Teffs and Tregs display distinct functional and metabolic profiles, orchestrated by key metabolic regulators such as pyruvate dehydrogenase (PDH) and phosphoglycerate kinase (PGK). Activated Teffs, including Th1 and Th17 subsets, depend predominantly on glycolysis and glutamine catabolism to support their rapid proliferation and pro-inflammatory responses (93, 102–104). Specifically, Th17 cells exhibit elevated PGK activity, which enhances glycolytic flux and the accumulation of glycolytic intermediates essential for their differentiation and function. In contrast, Tregs prioritize OXPHOS over glycolysis, facilitated by PDH-mediated entry of pyruvate into the tricarboxylic acid (TCA) cycle, thereby sustaining their immunosuppressive activities. This metabolic preference in Tregs is further supported by reduced expression of glycolytic enzymes and increased fatty acid oxidation, enabling them to utilize diverse energy substrates efficiently (105, 106).

The differential regulation by PDH and PGK not only delineates the metabolic pathways favoring Teffs and Tregs but also highlights potential therapeutic targets for modulating immune responses in cardiovascular diseases. Understanding these metabolic distinctions provides critical insights into maintaining the balance between pro-inflammatory and anti-inflammatory T cell populations, offering avenues for intervention in atherosclerosis and related pathologies.

Considering the metabolic characteristics of CD4^+^ T cells discussed above, the selection between glycolysis and glucose oxidation pathways emerges as a potential target for modulating the metabolism of CD4^+^ T cell subsets to control the inflammatory responses they trigger. Metabolic analysis shows that PDH is a key bifurcation point between glycolysis and glucose oxidation in T cells (107). PDH is inhibited by pyruvate dehydrogenase kinase (PDHK) (107). Specifically, PDHK1 is expressed in Th17 cells but not in Th1 cells, and its expression is low in Tregs. Inhibition or knockdown of PDHK1 selectively suppresses Th17 cells while increasing Tregs (107, 108).

PGK1, a key metabolic enzyme in the glycolytic pathway, could also be a potential target for regulating T cell function. Lu’s research found that in myocarditis, both glycolysis and PGK1 expression are elevated in cardiac CD4^+^ T cells and Th17 cells. Inhibition of PGK1 by NG52 reduced the cardiac damage caused by myocarditis and altered the infiltration patterns of CD4^+^ T cells, including Th17 cells, Th1 cells, and Tregs. NG52 also prevented the development of dilated cardiomyopathy (DCM). Mechanistically, NG52 blocks glycolysis and inhibits the phosphorylation of PDHK1, leading to increased accumulation of ROS in mitochondria and limiting the development of Th17 cells. Ultimately, NG52 inhibited the responses of CD4^+^ T cells and Th17 cells from patients with myocarditis. This study suggests that targeting PGK1 may be a promising approach for the treatment of ICI-induced myocarditis (90).

To better target CD4^+^ T cells, nanomaterials offer a powerful platform for enhancing the delivery and efficacy of PGK1 inhibitors like NG52 in treating myocarditis induced by ICIs. Functionalized nanoparticles, modified with ligands or antibodies that specifically recognize Th17-associated surface markers such as IL-17A receptor, CD4, or CCR6, enable precise targeting of glycolysis-dependent Th17 cells. Additionally, surface modifications using PEGylation enhance the systemic stability of nanoparticles, prolonging circulation time while reducing non-specific immune clearance. These nanoparticles encapsulate NG52, a potent PGK1 inhibitor, which disrupts glycolysis by blocking PGK1 activity, suppressing PDHK1 phosphorylation, and inducing mitochondrial ROS accumulation. This mechanism selectively impairs Th17 differentiation and inflammatory function, while sparing Tregs that rely on oxidative phosphorylation. The targeted and controlled release system ensures precise drug accumulation within inflamed cardiac tissues, significantly reducing off-target effects. This strategy achieves remarkable therapeutic outcomes in experimental myocarditis models. It reprograms CD4^+^ T cell subsets by reducing Th17 and Th1 infiltration while increasing Treg expansion, alleviates myocardial inflammation and fibrosis, and prevents progression to DCM. Moreover, nanomaterials improve the bioavailability and pharmacokinetic stability of NG52, allowing for lower doses and reduced systemic toxicity. By integrating nanotechnology with PGK1 inhibition, this approach preserves the anti-tumor efficacy of ICIs while minimizing irAEs, providing a refined and transformative strategy for treating ICI-induced myocarditis.

Besides, there are also lots of other strategies regulating the glycolytic process on the way. For example, 2-DG is a typical inhibitor of the glycolytic pathway by blocking hexokinase, the first enzyme of glycolysis. Treatment of T cells with 2-DG reduces glycolytic activity, leading to decreased IL-17 production while promoting Foxp3 induction (106). Additionally, the transcription factor HIF-1α is selectively expressed in Th17 cells, and its induction requires signaling via mTOR, a central regulator of cell metabolism. The mTOR inhibitor rapamycin can also block mTOR-dependent metabolic pathways to achieve a similar effect (109–111). Besides, a CTLA-4 agonist abatacept has been used as an antidote for life-threatening, glucocorticoid-refractory ICI–induced myocarditis (41). Mechanistically, it binds to CD80 and CD86 on antigen-presenting cells, blocks the engagement of CD28 on T cells and may downregulate mTOR pathway which relates to T-cell glycolysis, metabolism and activation (112). Several clinical trials including ClinicalTrials.gov number NCT05195645 and NCT05335928 involving patients with myocarditis are on the way. Overall, these results indicate that inhibition of glycolysis blocks the development of Th17 cells and promotes the generation of Tregs, protecting the body from autoimmune inflammation. Metabolic reprogramming targeting the glycolytic pathway, including targets such as PGK1 and PDHK1, may be a promising approach for ICI-induced cardiotoxicity while retaining anti-tumor efficacy.

Although metabolic reprogramming targeting glycolysis in T cells provides promising strategies to alleviate cardiovascular toxicities induced by ICIs, caution is necessary as these strategies might inadvertently impair anti-tumor immunity. Particularly, activated CD8^+^ T cells, which play a crucial role in tumor eradication, heavily rely on glycolysis to maintain their proliferation, cytokine secretion (e.g., IFN-γ, granzyme B), and cytotoxic functions (113). As demonstrated by Ho et al. (2015), interference with glycolytic metabolism—specifically inhibition of phosphoenolpyruvate (PEP) production—markedly impairs T cell receptor-induced calcium signaling and downstream NFAT-mediated transcription, ultimately diminishing the anti-tumor capability of CD8^+^ T cells (91, 113). Thus, metabolic interventions targeting glycolysis (such as using glycolytic inhibitors like 2-DG or 3PO) require careful dose optimization and precise targeting to achieve cardiovascular protective effects without compromising the essential anti-tumor immune responses. This balance should be a key consideration in future clinical studies and translational research.

#### 3.1.2 Immune reprogramming targeting KEY signaling pathway

**Figure 3:**
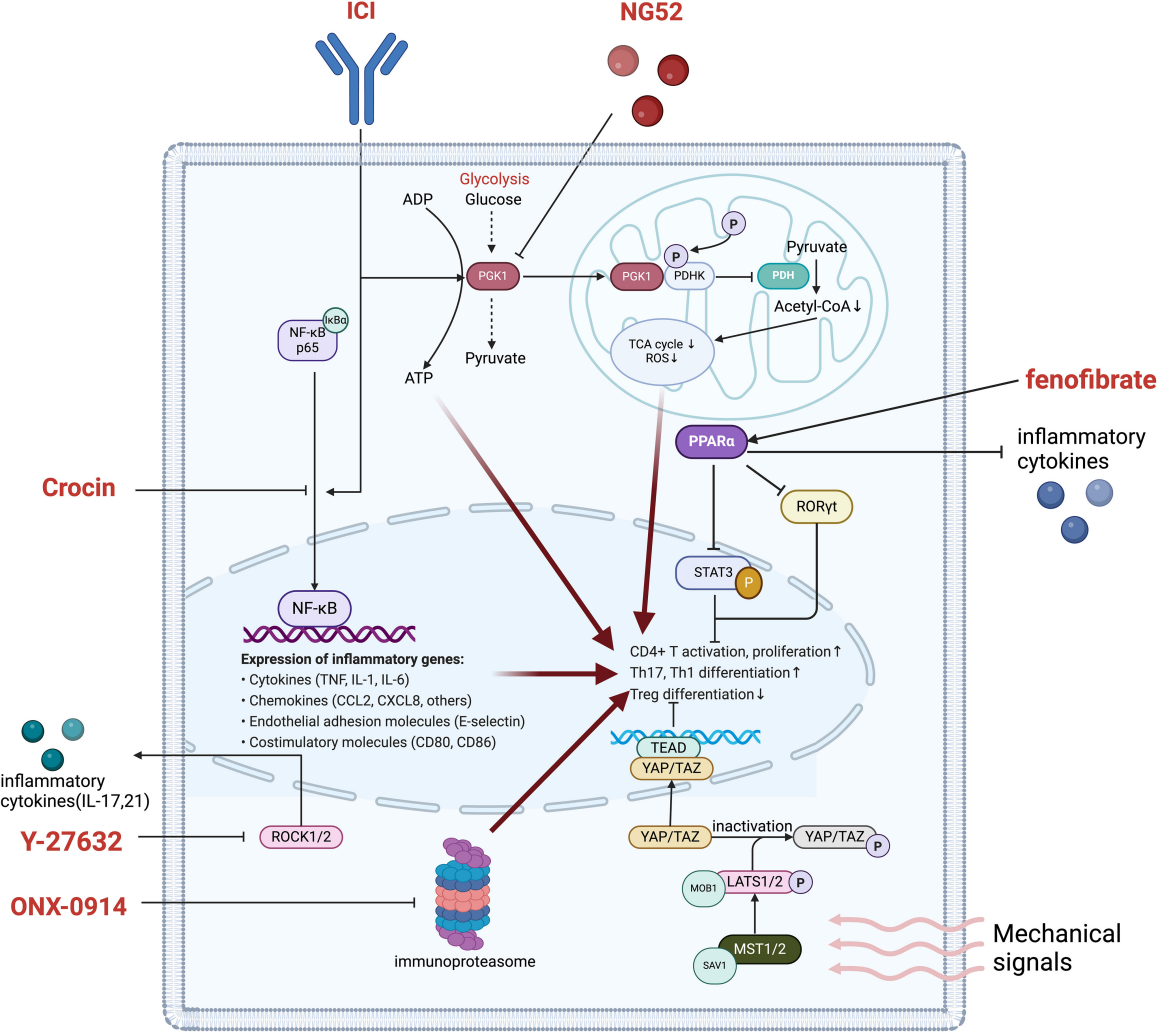
Main pathways in T cell regulation improving ICI-induced cardiovascular toxicity. phosphoglycerate kinase 1, PGK1; ADP, Adenosine Diphosphate; ATP, adenosine triphosphate. COA, Coenzyme A; TCA cycle, Tricarboxylic Acid cycle; ROS, Reactive Oxygen Species; PDHK, Pyruvate Dehydrogenase Kinase; PDH, Pyruvate Dehydrogenase; PPARα, Peroxisome Proliferator-Activated Receptor Alpha; RORγt, retinoic acid-related orphan receptor gamma t; STAT3, Signal Transducer and Activator of Transcription 3; TEAD, TEA Domain Transcription Factor; YAP, Yes-associated protein; TAZ, WWTR1, WW domain-containing transcription regulator 1; MOB1, MPS One Binder 1; LATS1/2, Large Tumor Suppressor 1/2; ROCK1/2, Rho-Associated Coiled Coil-Containing Protein Kinase 1/2; NF-κB, Nuclear Factor Kappa-B.

Numerous key signaling pathways—such as the HIPPO pathway, immunoproteasome, ROCK, NF-κB, and PPARα—play a central role in regulating T cell survival, function, and differentiation. These pathways not only influence the metabolic activities of T cells but also directly impact the balance between pro-inflammatory Th17 cells and anti-inflammatory Treg cells, which is crucial for maintaining immune homeostasis and preventing excessive inflammatory responses, as shown in **Figure 3** (114). By targeting these signaling pathways, the ratio of Th17 to Treg cells can be effectively modulated to achieve immune balance, thereby reducing irAEs such as cardiovascular toxicity induced by ICIs, without compromising their antitumor efficacy. This review systematically summarizes the mechanisms by which these signaling pathways contribute to immune remodeling and proposes targeting them through immune regulation as a potential therapeutic strategy, offering new directions for optimizing the safety and efficacy of ICI therapy.

##### 3.1.2.1 Immuno-reprogramming targeting the HIPPO pathway

In immune reprogramming, the role of the HIPPO pathway in regulating T cell function and immune responses is crucial. This pathway is not only involved in the regulation of cell proliferation and apoptosis, but also plays a central role in the activation and differentiation of immune cells. Studies have shown that key components of the HIPPO signaling pathway, such as Mammalian STE20-like kinase 1 (Mst1/2) and transcriptional coactivator with PDZ-binding motif (TAZ), have dual regulatory effects on immune responses and inflammatory responses (115). Specifically, the activity of Mst1/2 is associated with the maintenance of T cell homeostasis, and its deletion may promote the differentiation of Th17 cells. TAZ, on the other hand, further promotes the proliferation of Th17 cells and inhibits the generation of Treg cells by regulating retinoic acid-related orphan receptor gamma t (RORγt) (116, 117). This regulatory mechanism means that targeting the HIPPO signaling pathway can precisely regulate the balance between pro-inflammatory Th17 cells and anti-inflammatory Treg cells, thereby playing an important role in the treatment of ICI-related cardiovascular toxicity.

The HIPPO signaling pathway is significantly activated in heart CD4+ T cells in ICI-related myocarditis (118, 119). ICI-related myocarditis reduces Mst1 kinase activity and activates TAZ, which acts as a co-activator of RORγt to promote Th17 cell differentiation and inhibit Treg cell development, TEA domain transcription factor 1 (TEAD1) suppresses TH17 differentiation and promotes Treg cell development by inhibiting the function of TAZ (117). This indicates that HIPPO signaling activation and TEAD negatively regulate TAZ-mediated Th17 differentiation. Similar experiments showed that TEAD1 has a higher affinity for TAZ than RORγt or FOXP3 and can disrupt the interaction between TAZ and RORγt or FOXP3. Moreover, TEAD1 significantly reduces Th17 activity mediated by TAZ or RORγt. In contrast, strong TEAD1 expression separates TAZ from RORγt and FOXP3, actively promoting Treg cell differentiation (107, 116, 120). Studies above identify the significance of HIPPO pathway in the immune reprogramming strategies towards ICI-induced myocarditis.

It has been reported that the mechanical trafficking of cytokines within lymphocytes, which play an important role in the inflammatory process, can be regulated by mechanical waves (121, 122). Low-intensity pulsed ultrasound (LIPUS), a novel non-invasive therapeutic approach, has emerged as a promising method for treating cardiovascular diseases by leveraging this mechanism. Preclinical studies in murine models have shown its efficacy in improving ischemia-induced cardiac dysfunction, reducing angiotensin II-mediated myocardial fibrosis, and attenuating left ventricular remodeling after myocardial infarction (123, 124). Compared with the PD-1 inhibitor group, LIPUS treatment increased the expression of Mst1 and TEAD-1 and decreased the expression of TAZ. These results suggest that LIPUS may regulate autoimmune inflammation by downregulating the core kinase Mst1 in the HIPPO pathway, and regulating the mutual differentiation of Treg and Th17 cells by altering the interaction between the transcription factors FOXp3 and RORγt via the Mst1-TAZ axis (116). LIPUS therapy can improve immune imbalance and relieve cardiac immune inflammation and heart failure caused by PD-1 inhibitors by mediating the mechanical transmission and regulation of the downstream HIPPO pathway of CD4+ T cells (116, 125). Therefore, LIPUS therapy may represent a promising non-invasive treatment strategy for ICI-related myocarditis which is a serious condition that can arise from ICIs therapies. However, it is important to note that clinical trials specifically investigating the efficacy and safety of LIPUS therapy for heart disease are currently not yet available; thus, a well-designed prospective cohort study should be conducted first (126).

##### 3.1.2.2 Immune reprogramming targeting immunoproteasome

Immunoproteasome is a variant of proteasome with structural differences in 20S subunits. Commonly, the proteasome degrades proteins into smaller peptides that can then be displayed on the cell surface to alert immune cells (127). The variation of immunoproteasome makes it optimized for the production of antigenic peptides with higher binding affinity to MHC-I molecules (128). Apart from antigen presentation, immunoproteasome is also responsible for maintaining protein homeostasis and regulating signaling pathways (129). Besides, the immunoproteasome plays a critical role in T cell expansion, cytokine production, and T helper cell differentiation, suggesting the potential to alter immune status (130). As a result, strategies targeting immunoproteasomes can reprogram metabolism by affecting protein metabolism.

In fact, previous research has reported inhibition of the immunoproteasome ameliorated disease symptoms in different animal models for autoimmune diseases. In an animal model, Bockstahler et al. demonstrated that the immunoproteasome promotes a proinflammatory immune response dominated by Th17 and Th1 cells while impairing the function of Tregs in ICIs-induced myocarditis). The study found that inhibiting key immunoproteasome subunits LMP2 and LMP7, or administering the immunoproteasome inhibitor ONX 0914, significantly reduced cardiac inflammation and fibrosis, leading to improved heart function (131). Immunoproteasome inhibition restored immune balance by reducing the activity of Th17 and Th1 cells, suppressing the production of pro-inflammatory cytokines, and promoting the proliferation of Tregs (132, 133). Furthermore, treatment with ONX 0914 diminished the pro-inflammatory response of monocytes activated via the Toll-like receptor (TLR) signaling pathway, thereby further alleviating ICI-induced autoimmune myocarditis (134). These findings suggest that targeting the immunoproteasome could serve as a potential therapeutic strategy for ICI-related irAEs by inhibiting pro-inflammatory responses and enhancing immune regulation. However, clinical trials are necessary to further verify the safety and efficacy of this therapy in humans (135).

##### 3.1.2.3 Immune reprogramming targeting the ROCK pathway

Rho kinase (ROCK), a downstream effector of Rho GTPase, has been demonstrated to be involved in cell adhesion, motility, and contraction (136). Indeed, ROCK is well-known for its involvement in the tumor cell and tumor microenvironment, including ability to enhance tumor cell progression, migration, metastasis, and extracellular matrix remodeling. Notably, ROCK is also considered to modulate the function of immune cells, including dendritic cells (DCs), macrophages, natural killer cells and T cells (137). Besides, inhibition of ROCK was shown to alleviate the pathogenesis of immunopathogenic diseases. It has been proved that ROCK2 participates in the differentiation of Th17 cells, regulating inflammatory responses in autoimmune disorders through the JAK/STAT pathway (138).

The RhoA/ROCK signal pathway is located upstream of HIF-1α. The pro-fibrotic effect of HIF-1α is negatively regulated by Notch3 through the RhoA/ROCK/HIF-1α signal pathway (139). The Notch pathway has been shown to play a key role in mammalian heart development. After myocardial injury, Notch1, Hes1 and Jagged1 in the heart significantly increase, indicating that the Notch signaling pathway is involved in the regulation of myocardial injury (140). The various effects of Notch signal transduction include inducing stem cell differentiation, promoting neovascularization, alleviating myocardial fibrosis and reducing cardiomyocyte apoptosis (141–146). In addition, it has also been shown that inhibiting ROCK activity effectively alleviates the upregulation of IL-1β caused by activation of the Notch signal pathway (147). IL-1 signaling activates innate immune cells including antigen presenting cells, and drives polarization of CD4+ T cells towards T helper type (Th) 1 and Th17 cells (148). As a result, the inhibition of ROCK pathway further slows the development of inflammation. Therefore, a deeper understanding of ROCK signal transduction in different cell types and the interactions between the ROCK signal pathway and other pathways may help develop more innovative and precise targeted therapies to provide patients with better clinical outcomes.

Inhibition of the ROCK pathway shows significant therapeutic potential in regulating immune and alleviating immune-related myocarditis induced by ICIs (149). Li et al. demonstrated that Y-27632, a ROCK inhibitor, effectively downregulates the expression of the pro-inflammatory factor IL-1β by inhibiting the Notch and TLR signaling pathways, thereby reducing cardiac inflammation and fibrosis in experimental autoimmune myocarditis (EAM) (147). Research has shown that the ROCK pathway is closely linked to various immune response processes. Intervention with Y-27632 significantly improved cardiac function, reduced the heart-to-body weight ratio, and decreased the number of monocytes in the spleen, indicating its effectiveness in alleviating systemic inflammation (147, 150).

In the EAM mouse model, treatment with Y-27632 not only significantly reduced the expression of Notch signaling-related genes such as IL-1β, Notch1, and Hes1, but also inhibited the activity of TLR2, thereby controlling the pro-inflammatory immune response (147). Inhibition of the ROCK pathway ameliorated ICI-induced myocardial injury by regulating immune status, particularly by reducing the production of pro-inflammatory cytokines. This immune reprogramming strategy suppressed the activity of Th17 cells and other pro-inflammatory cells, restoring immune homeostasis and thus alleviating myocarditis symptoms and improving prognosis (151, 152).

In summary, immune reprogramming by targeting the ROCK pathway offers a promising therapeutic strategy for ICI-related irAEs. Inhibiting the ROCK pathway can effectively reduce the pro-inflammatory immune response without affecting anti-tumor immunity, providing a new direction for the clinical treatment of ICI-related myocarditis. However, further research and clinical validation remain critical steps in assessing the safety and efficacy of this therapy.

##### 3.1.2.4 Immune reprogramming targeting NF-κB pathway

NF-κB plays a central role in the pathogenesis of ICI-related myocarditis, a severe irAEs. Overactivation of the NF-κB pathway drives excessive production of pro-inflammatory cytokines such as interleukin-1β (IL-1β), interleukin-6 (IL-6), and tumor necrosis factor-alpha (TNF-α), leading to immune cell infiltration, myocardial damage, and fibrosis (153–155). Targeting NF-κB through immune reprogramming offers a promising strategy to mitigate these effects while preserving the antitumor efficacy of ICIs.

Recent studies highlight the potential of this approach. Horiguchi et al. demonstrated that angiopoietin-like protein 2 (ANGPTL2)-mediated activation of NF-κB contributes to immune imbalance by promoting Th17 cell differentiation, thereby worsening myocarditis. Inhibiting NF-κB can disrupt this cascade (156). Additionally, Zhang et al. (2022) showed that crocin reduces NF-κB activation and NOD-, LRR- and pyrin domain-containing protein 3 (NLRP3) inflammasome-mediated pyroptosis in ICI-related myocarditis, alleviating inflammation and cardiac injury (157). Besides, a recent clinical study has reported potential of tocilizumab, an inhibitor of NF-κB-derived IL-6, for refractory severe ICI-induced myocarditis, indicating promising application of NF-κB-associated therapies (158).

These findings support targeting NF-κB as a viable therapeutic strategy to reduce ICI-induced myocarditis and other inflammatory irAEs, balancing immune modulation without compromising cancer therapy.

##### 3.1.2.5 Immune reprogramming targeting PPARα

Peroxisome proliferator-activated receptor α (PPARα) is a ligand-activated transcription factor belonging, together with PPARγ and PPARβ/δ, to the NR1C nuclear receptor subfamily (159). Studies have demonstrated its effect in balancing the ratio of Th17 cells and Treg cells to regulate inflammation. As we discussed above, achieving a proper balance between Th17 and Treg cells is crucial for maintaining immune homeostasis. In diseases like cardiovascular disorders, an overactive Th17 response can amplify inflammation, whereas Treg cells serve to counterbalance this effect, supporting tissue repair and healing (160). This delicate equilibrium is vital for proper immune regulation.

Recent research has reported the unique performance of PPARα in this. First of all, it plays a crucial role in modulating immune responses by suppressing pro-inflammatory Th17 cell differentiation through targeting the IL-6/STAT3/RORγt pathway (161, 162). Th17 cells, which produce IL-17, are central to the development of autoimmune myocarditis and contribute to ICI-related myocarditis. Activation of PPARα, achieved through agonists such as fenofibrate, inhibits STAT3 phosphorylation and reduces RORγt expression, thereby suppressing Th17 cell differentiation and lowering IL-17 production, resulting in a reduced pro-inflammatory response in the myocardium (163, 164). Apart from suppressing Th17 cells, PPARα activation also promotes Treg function, restoring immune homeostasis by enhancing anti-inflammatory mechanisms (165). This balance between reducing harmful Th17 activity and promoting Treg function makes PPARα an effective target for treating both autoimmune and ICI-related myocarditis. By reprogramming immune status through the PPARα pathway, myocardial inflammation and fibrosis can be mitigated, addressing the severe cardiac complications seen in ICI-induced myocarditis (163). PPARα’s ability to modulate the immune system without compromising anti-tumor immunity makes it a promising candidate for managing irAEs associated with ICI therapy.

### 3.2 Macrophage reprogramming therapy for ICI-induced cardiovascular events

Macrophages are highly plastic cells that can polarize into two major phenotypes, M1 (pro-inflammatory) and M2 (anti-inflammatory), depending on the microenvironment (166). These phenotypes exhibit significant differences in their metabolic pathways and functions. M1 macrophages primarily rely on glycolysis for their energy metabolism, characterized by lower mitochondrial function (166). Glycolysis not only supplies M1 macrophages with rapid energy but also promotes the inflammatory response through metabolic by-products such as succinic acid and ROS (167). These cells drive chronic inflammation in the arterial wall by secreting pro-inflammatory cytokines like TNF-α, IL-1β, and IL-6, which contribute to the formation and progression of atherosclerotic plaques (13, 18, 91). Additionally, M1 macrophages are involved in lipid uptake and foam cell formation, further exacerbating atherosclerosis (168, 169). In contrast, M2 macrophages depend on fatty acid oxidation (FAO) and OXPHOS for their energy needs, displaying higher mitochondrial activity (167). This metabolic profile supports their anti-inflammatory roles, facilitating tissue repair and promoting plaque stability. M2 macrophages secrete anti-inflammatory factors like IL-10, which help to stabilize plaques by reducing inflammation and encouraging fibrosis, thereby decreasing the risk of plaque rupture (13, 24, 25). Modulating the phenotypes of macrophages, particularly by promoting a shift from the M1 to M2 phenotype, has shown promise in reducing atherosclerotic progression and enhancing plaque stability.

**Figure 4:**
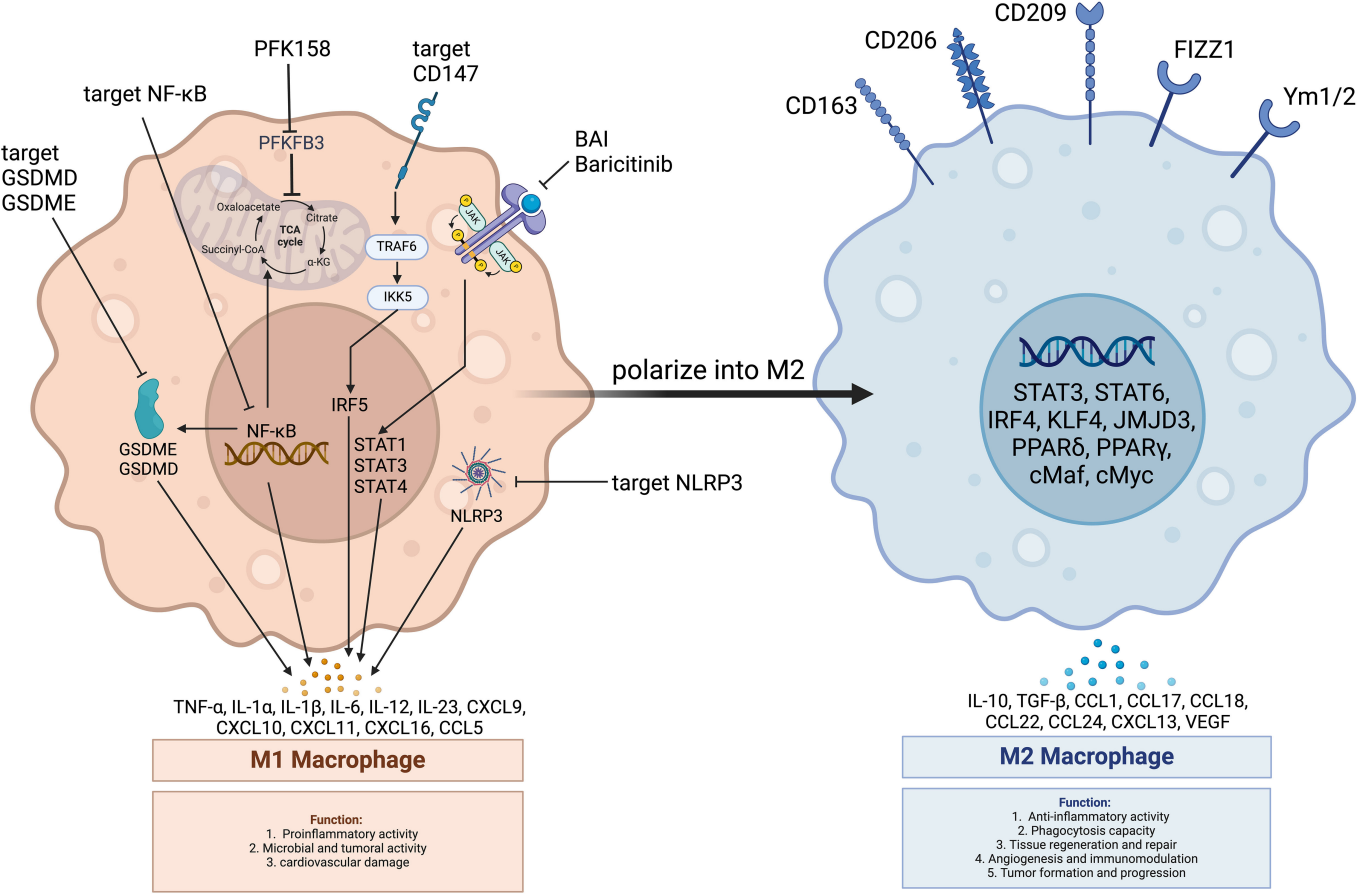
Main pathways in macrophage regulation improving ICI-induced cardiovascular toxicity. PFK158: PFKFB3 inhibitor; PFKFB3: 6-Phosphofructo-2-kinase/Fructose-2,6-bisphosphatase 3; GSDME: Gasdermin E; GSDMD: Gasdermin D; JAK: Janus Kinase; STAT3: Signal Transducer and Activator of Transcription 3; STAT6: Signal Transducer and Activator of Transcription 6; NF-κB: Nuclear Factor kappa B; NLRP3: NOD-like Receptor Family Pyrin Domain Containing 3; TRAF6: Tumor Necrosis Factor Receptor-Associated Factor 6; IKK: Inhibitor of κB Kinase; IRF5: Interferon Regulatory Factor 5; IRF4: Interferon Regulatory Factor 4; PPARα: Peroxisome Proliferator-Activated Receptor Alpha; PPARγ: Peroxisome Proliferator-Activated Receptor Gamma; Baricitinib: A Janus Kinase Inhibitor; CD147: Cluster of Differentiation 147; CD206: Cluster of Differentiation 206 (Macrophage Mannose Receptor); CD163: Cluster of Differentiation 163; CD209: Cluster of Differentiation 209 (DC-SIGN); Ym1/2: Chitinase-Like Proteins Ym1 and Ym2; FIZZ1: Found in Inflammatory Zone 1; CXCL: CXC Chemokine Ligand; CCL: CC Chemokine Ligand; IL-4: Interleukin 4; IL-13: Interleukin 13; IL-10: Interleukin 10; TGF-β: Transforming Growth Factor-beta; VEGF: Vascular Endothelial Growth Factor; cMyc: Myelocytomatosis Viral Oncogene Homolog; cMaf: Musculoaponeurotic Fibrosarcoma Oncogene Homolog; JMJD3: Jumonji Domain-Containing Protein 3; KLF4: Kruppel-Like Factor 4; TCA Cycle: Tricarboxylic Acid Cycle; α-KG: Alpha-Ketoglutarate; Succinyl-CoA: Succinyl-Coenzyme A; Citrate: Citric Acid.

Given the role of macrophages in the inflammatory processes of atherosclerosis and myocarditis, their plasticity offers a strategic target for therapeutic intervention in irAEs such as those induced by ICI, as shown in **Figure 4**.

#### 3.2.1 Immune reprogramming targeting glycolysis in macrophage

Recent research has underscored the significance of targeting the glycolytic pathway in macrophages to mitigate inflammation. Activated M1 macrophages undergo a metabolic shift towards glycolysis, a process regulated by enzymes like 6-phosphofructo-2-kinase/fructose-2,6-bisphosphatase 3 (PFKFB3), which enhances glycolytic flux to meet the energy demands of inflammation (98). Enhanced PFKFB3 expression is frequently observed in pro-inflammatory macrophages within atherosclerotic plaques, correlating with increased plaque instability and inflammation (170). Besides, glycolysis supports the rapid production of ATP and biosynthetic intermediates, facilitating the release of inflammatory cytokines that sustain a heightened immune response in inflamed tissues such as the arterial wall and myocardium (170).

Therapeutic inhibition of PFKFB3 has emerged as a potential strategy to regulate macrophage-driven inflammation in these contexts. Studies utilizing PFK158, a selective inhibitor of PFKFB3, have shown that partial suppression of glycolysis can significantly reduce macrophage-induced inflammation while maintaining some of their essential immune functions (98, 100). In experimental models of atherosclerosis, treatment with PFK158 led to a reduction in glycolytic activity of macrophages and peripheral blood mononuclear cells (PBMCs), which in turn decreased necrotic core size, reduced apoptosis within plaques, and resulted in a thicker fibrous cap—key markers of plaque stability (98). This suggests that suppressing glycolysis can shift the balance of macrophage activity away from an M1-like pro-inflammatory state towards a more M2-like, reparative profile, ultimately reducing tissue damage and stabilizing plaques (98).

Mechanistically, PFKFB3 inhibition downregulates key glycolytic regulators such as HIF-1α and glucose transporters like glucose transporter 3(GLUT3), which are critical for maintaining the heightened metabolic demands of M1 macrophages (98, 171). This shift leads to a reduction in pro-inflammatory signaling and encourages a more balanced macrophage response (171). Moreover, glycolysis inhibition may enhance autophagy in macrophages, promoting the clearance of cellular debris and further supporting tissue repair (98, 100).

Overall, the ability of macrophages to undergo metabolic reprogramming presents a promising therapeutic avenue for managing ICI-induced irAEs, particularly myocarditis and atherosclerosis. By targeting metabolic enzymes like PFKFB3 to modulate glycolysis, it is possible to reduce the inflammatory potential of macrophages while preserving their reparative functions. This dual effect offers a balanced approach for mitigating the cardiovascular risks associated with ICI therapy, paving the way for improved management of cancer treatment-related side effects.

#### 3.2.2 Immune reprogramming targeting the GSDM family

The gasdermin (GSDM) family, including GSDMA, GSDMB, GSDMC, GSDMD, GSDME (also known as DFNA5) and DFNB59 (also known as PJVK), has emerged as a crucial player in mediating pyroptosis. Pyroptosis is a form of inflammatory cell death characterized by cell membrane pore formation and the release of pro-inflammatory cytokines such as IL-1β and IL-18 (172, 173) which is particularly relevant in ICI-induced myocarditis and atherosclerosis (174, 175). Given the GSDM family’s role in driving inflammation, targeting their activity through reprogramming presents a promising therapeutic approach.

GSDME is known to be highly expressed in M1 macrophages within atherosclerotic plaques, promoting pyroptosis and furthering inflammation. Pyroptosis, unlike other forms of cell death, results in cell lysis and the release of inflammatory mediators, which can amplify local immune responses and worsen tissue damage (174, 176). In detail, GSDMD activation leads to mitochondrial rupture and mtDNA leakage, which in turn activates the stimulator of interferon genes (STING)- interferon regulatory factor 3 (IRF3)/NF-κB axis to mediate atherosclerosis progression (22). In addition, GSDME can be cleaved by caspase 3 to the form membrane pores by the N-terminal fragment of GSDME which may lead to release of inflammatory cytokines like IL-1β and TNF-α and convert non-inflammatory apoptosis into inflammatory pyroptosis (177, 178). Therefore, modulating the metabolic context that activates caspase 3 and GSDME could shift macrophages away from a pro-inflammatory state toward more controlled forms of cell death, such as apoptosis (179). Zhai et al. have pretreated THP-1 cell (a monocyte cell line) -derived macrophages with a caspase 3 specific inhibitor (Z-DEVD-FMK) for 1 hour, followed by treatment with TNF for 36 hours. As a result, Z-DEVD-FMK treatment decreased the expression of activated caspase 3, GSDME, and GSDME-N and reduced the induction of pyroptosis in THP-1 cell-derived macrophages (180). Besides, the research silenced GSDME with siRNAs in THP-1 cell-derived macrophages and then then reported that the silencing resulted in decreased expression of GSDME-N and reduction in TNF-induced pyroptosis (180).

Another important regulatory axis is the STAT3-GSDME pathway. STAT3, a transcription factor that responds to inflammatory stimuli, upregulates GSDME expression, thus enhancing the propensity for pyroptosis in macrophages (181). By targeting the caspase 3/GSDME pathway, it might reduce macrophage pyroptosis and the subsequent release of damaging inflammatory mediators. This approach could also help in reprogramming the immune response in the heart during ICI-induced myocarditis, helping to limit the extent of cardiac inflammation while maintaining some level of immune surveillance (182). Besides, the function of STAT3-GSDME pathway suggests that inhibiting STAT3, or the upstream metabolic pathways that influence its activity, could reduce GSDME levels and thus diminish pyroptosis-driven inflammation. Such interventions could be particularly relevant in mitigating the overactive immune responses seen in ICI-induced myocarditis, where macrophage-driven inflammation contributes to cardiac injury (174, 181).

Therefore, several studies have suggested the potential benefits of targeting GSDME to treat inflammation, especially atherosclerosis. For instance, genetic deletion of GSDME in GSDME−/−/apolipoprotein E (ApoE)−/− mouse models led to smaller atherosclerotic lesions and reduced levels of inflammatory cytokines such as IL-1β and MCP-1 (174, 176). These findings suggest that reducing GSDME activity can stabilize the inflammatory environment within vascular lesions, offering a pathway for controlling inflammation without completely suppressing immune function (174, 176). Recent studies have found that GSDMD-deficient mice have reduced atherosclerotic plaque area in ApoE-/- mice induced by a high-fat diet (174). Furthermore, results from single-cell RNA sequencing showed that the transcriptional factor activities of NF-κB and IRF3 were reduced in GSDMD-deficient mice. The study also found that the GSDMD-specific inhibitor GI-Y1 can effectively reduce the progression of atherosclerosis (22). Therefore, targeting the specific inhibitory drugs of GSMDM can become an important research direction for reducing the progression of atherosclerosis.

However, the challenge lies in selectively targeting these pathways to reduce the inflammatory impact without impairing the beneficial aspects of immune activation. Precision strategies that focus on the regulation of GSDMs within specific immune cell populations may provide a way forward, allowing for the attenuation of irAEs while preserving the anti-tumor effects of ICIs (183). Given the growing recognition of the role of pyroptosis in various inflammatory diseases, targeting GSDMs presents a novel and promising direction in the development of therapies for managing ICI-induced adverse events.

#### 3.2.3 Immune reprogramming targeting the JAK/STAT pathway

The JAK/STAT pathway mainly mediates the signaling of cytokine receptors (184). In the field of immune reprogramming, the JAK/STAT pathway stands out as a pivotal regulator of macrophage polarization, particularly in balancing pro-inflammatory M1 and anti-inflammatory M2 macrophages (53). Activation of STAT3 enhances the expression of anti-inflammatory mediators such as IL-10 and arginase-1(Arg-1), facilitating the shift toward M2 macrophages and dampening the pro-inflammatory activity of M1 macrophages (184). In contrast, STAT1 activation is primarily associated with M1 polarization, driving the expression of pro-inflammatory cytokines like TNF-α, IL-6, and IL-1β (53, 185). For example, IFN-γ binds to its receptor and activates JAK, thus inducing the phosphorylation of STAT1, which leads to the polarization of macrophages to M1 (186). In addition, the suppressor of cytokine signaling (SOCS) is a feedback inhibitor of JAK/STAT signaling. It was found that the deficiency of SOCS1 and SOCS3 promoted M1 macrophage polarization by activating the JAK1/STAT1 signaling pathway (187). Further research showed that increased phosphorylation of STAT3 could feedback inhibit the expression of STAT1 by upregulating the expression of SOCS3, thereby inhibiting macrophage polarization towards M1 phenotype (188, 189). Thus, the JAK/STAT pathway plays a dual role in macrophage reprogramming, acting as a crucial switch for modulating macrophage function.

In fact, in the experimental EAM model, Baicalein (5,6,7-trihydroxyflavone, C+15H10O5, BAI), a primary bioactive compound with potent anti-inflammatory properties derived from the Scutellaria baicalensis root, have exerted good therapeutic effects against various autoimmune diseases. Mechanically, it demonstrated that BAI alleviates M1/Th1-secreted TNF-α- and IFN-γ-induced cardiomyocyte death in EAM mice by inhibiting the JAK-STAT1/4 signaling pathway (190). Besides, in models of diseases such as atherosclerosis and ICI-induced myocarditis, drugs targeting the JAK1/STAT3 pathway, like Baricitinib, have been shown to reduce inflammation by driving macrophages toward an M2 anti-inflammatory phenotype (53). At present, JAK inhibitors have been put into clinical application. Nguyen et al. reversed a case of nearly lethal ICI-myocarditis by using specific patient-dose adjusted abatacept combined with ruxolitinib (a JAK inhibitor) (Trial registration number NCT04294771) (39). In addition, Usui reported possibilities that the administration of baricitinib, a JAK inhibitor, was effective in a case of fulminant myocarditis with COVID‐19 infection, which may serve as basis for treatment of patients with severe ICI-induced cardiovascular events (191).

In summary, targeting the JAK/STAT pathway is a promising strategy for macrophage reprogramming, effectively balancing M1/M2 phenotypes to mitigate inflammation and promote tissue repair. However, further clinical studies are needed to validate the safety and efficacy of JAK/STAT-targeted therapies in conditions like ICI-induced myocarditis. Preclinical research should optimize the specificity of JAK inhibitors and STAT modulators, while basic studies should investigate the pathway’s interaction with macrophage metabolism and identify biomarkers for real-time monitoring. These efforts, combined with advanced delivery systems, can enhance precision and therapeutic outcomes.

#### 3.2.4 Other pathways in macrophage reprogramming

Apart from strategies mentioned above, there are also other significant pathways for reprogramming macrophages (**Table 1**). For example, complementary pathways act as auxiliary regulators, further reducing M1-mediated inflammation and promoting the anti-inflammatory functions of M2 macrophages. Together, these pathways offer promising targets for treating a range of inflammatory diseases.

**Table 1:** Other potential pathway in macrophage regulation against myocarditis and atherosclerosis.

Potential Pathway	Potential Target	Function	Ref.
SMAD4 signaling pathway	TREM2	Maintain metabolic homeostasis of macrophage; Participate in phagocytosis and glycometabolism	(192–195)
Cyclin-dependent kinase pathway	p27^kip^	Inhibit macrophage proliferation by blocking cell-cycle progression	(192, 196)
Cyclin-dependent kinase pathway	SR-A1	Trigger for macrophage expansion and further plaque expansion	(192, 197)
Cdkn 2a pathway	Human 9p21 locus	Regulate monocyte/macrophage proliferation	(198)
CSF-1/CSF-1R pathway	csCSF-1, GM-CSF	Contribute to macrophage proliferation, survival in lesions, and expansion in atherosclerosis	(192, 199, 200)
MAPK signal transduction	Irgm1	Affect macrophage apoptosis by regulating JNK/p38/ERK phosphorylation	(201)
mTOR and OxPhos pathway	AMPK	Inhibit mTOR, promote OxPhos and mitochondrial biogenesis, facilitating memory T cell differentiation rather than cytotoxic CD8+ T cell	(202–205)
Glycolysis and OxPhos pathway	MCT-1	Accumulated lactic acid promotes OxPhos and suppresses cytotoxic CD8+ T cell proliferation and cytokine production	(202, 206, 207)
NRF2 pathway	GSH	Detoxify ROS, and support activation-induced glycolytic metabolic reprograming in T cells	(202, 208, 209)
Pentose phosphate pathway (PPP)	Pck1	Catalyze the production of G6P, which enters the PPP and produces NADPH to enhance the survival of memory CD8+ T cells	(210, 211)
LDLR/STAT3/ROR-γt pathway	PCSK9	Promote Th17 cell differentiation, elevate IL-17 levels, and aggravate inflammation in myocarditis	(212)
4-1BB pathway	4-1BB/4-1BBL	Suppress T cell proliferation and Th1-type cytokines production	(213)

NF-κB signaling pathway is a key regulator of macrophage-mediated inflammation. NF-κB activation promotes M1 macrophage polarization, enhancing glycolysis and amplifying pro-inflammatory signals such as IL-1β and TNF-α (214). Inhibiting NF-κB not only suppresses M1 polarization but also facilitates M2 polarization, thereby boosting anti-inflammatory responses. For example, in the context of PAPP-A inhibition, the combined suppression of NF-κB significantly reduced inflammation and promoted M2 macrophage polarization, offering protection against atherosclerosis (215).

Besides, the NLRP3 inflammasome plays a central role in macrophage-driven inflammation, particularly in M1 macrophages (216). Metabolic stress, such as mitochondrial dysfunction and increased glycolysis, can activate the NLRP3 inflammasome, which further drives pro-inflammatory responses (217). Targeting NLRP3 to suppress its activity has been shown to reduce M1 macrophage-driven inflammation and, when combined with JAK/STAT3 activation, enhances the phenotype shift toward M2 macrophages (215).

Not like the central role of the JAK/STAT pathway in macrophage polarization, additional mechanisms like TRAF6 (tumor necrosis factor receptor-associated factor 6)-IKK (inhibitor of κB kinase)-IRF5 (IFN regulatory factor 5) signaling and exosomal communication offer important insights into immune regulation. CD147, for instance, drives the M1 pro-inflammatory phenotype via the TRAF6-IKK-IRF5 axis, while also impairing efferocytosis, a key process for resolving inflammation. Targeting CD147 not only reduces inflammation but also enhances efferocytosis, showing promise in diseases like atherosclerosis (218).

Moreover, exosomal signaling between macrophages and other cells adds another layer of complexity. In PD-1 inhibitor-induced cardiac dysfunction, macrophage-derived exosomes carrying miR-34a-5p promote cardiomyocyte senescence and injury. Inhibiting miR-34a-5p in macrophages mitigates this damage, highlighting the potential for exosomal-targeted therapies (219).

In summary, targeting pathways such as NF-κB, NLRP3 inflammasome, TRAF6-IKK-IRF5, and exosomal signaling offers a multifaceted approach to reprogramming macrophages and mitigating inflammation. However, challenges remain, including pathway-specificity and limited clinical translation of novel strategies like exosomal therapies. Future efforts should prioritize precision delivery systems, such as nanotechnology and biomimetic carriers, to enhance specificity while minimizing off-target effects. Additionally, integrating real-time monitoring of macrophage phenotypes and metabolic states with pathway-specific interventions may optimize therapeutic outcomes. These advancements hold the potential to redefine the management of inflammatory and immune-related diseases through personalized and pathway-targeted treatments.

## 4 Conclusion

The extensive utilization of ICIs in oncological therapy is associated with an elevated incidence of adverse cardiovascular events, including acute myocarditis and chronic atherosclerosis. ICI enhance the anti-tumor activity of T cells by blocking the PD-1/PD-L1 or CTLA-4 pathway, thereby relieving immunosuppression. However, this overactivation of T cells may also result in the attack of normal tissues, such as the heart, which can lead to the development of acute myocarditis. Pro-inflammatory factors released by T cells (such as IFN-γ and TNF-α) exacerbate myocardial inflammation and damage cardiomyocytes through the induction of oxidative stress. In the context of atherosclerosis, ICI-activated T cells and macrophages are responsible for driving the inflammatory response through the glycolytic pathway. Macrophages of the M1 phenotype secrete pro-inflammatory factors and form foam cells, which contribute to the formation and instability of arterial plaques. Further investigation is essential to deepen our understanding of the mechanisms behind myocarditis. One key question is why some patients develop myocarditis while others do not. Factors such as genetic predisposition, specific autoantibodies, and pre-existing health conditions might influence this variability, but more research is needed to confirm these connections. Additionally, while it is known that T cell overactivation significantly contributes to myocarditis, the exact immune pathways and cellular interactions involved are still not fully understood. For example, the roles of B cells, autoantibodies, and other immune system components in myocarditis are subjects of ongoing research. Another challenge is the lack of identifiable biomarkers for the early detection of ICI-induced myocarditis, which complicates the prediction and diagnosis of this condition before it manifests. Gaining a better understanding of these mechanisms could help identify patients at higher risk and improve monitoring for those receiving ICI therapy. Lastly, the interactions between immune and non-immune cells, such as endothelial cells and cardiac fibroblasts, in the context of myocarditis are not well understood. These interactions may play a significant role in the development of inflammation, fibrosis, and long-term cardiac damage. Further research is required to fully elucidate the immune pathways involved in ICI-induced myocarditis. However, current understanding highlights the importance of managing T cell activation and controlling inflammation in the prevention and treatment of this serious irAE.

Immune reprogramming has the potential to be a valuable therapeutic strategy for the modulation of these cardiovascular complications, particularly through the regulation of pathways in T cells and macrophages. The targeting of glycolysis in T cells and fatty acid oxidation in macrophages has been demonstrated to be an effective method of reducing the inflammation and tissue damage caused by excessive immune responses. Nevertheless, research on these mechanisms still has numerous uncharted territories.

Firstly, although the initial association between T cell metabolism and myocarditis has been confirmed, further study is required to ascertain the effect of glycolysis inhibition on long-term immune responses. In particular, it is necessary to determine how to precisely inhibit the pro-inflammatory response in the heart without weakening antitumor immunity. The intricacies of macrophage regulation in atherosclerosis remain poorly understood, particularly the impact of the dynamic alterations in macrophage subtypes within atherosclerotic plaques at distinct stages of disease progression. Furthermore, the role of the interaction between autophagy and lipid metabolism in the formation of foam cells and the stability of atherosclerotic plaques requires further analysis.

It is recommended that future studies employ multi-omics technologies to elucidate the dynamic changes in immune status during cardiovascular toxicity and to develop personalized treatment regimens in combination with real-time monitoring of metabolic markers. The combination of metabolic regulation with traditional cardiovascular protective therapies, along with in-depth research on ROS production, mitochondrial function and autophagy pathways, and the development of new delivery systems, such as biocompatible materials and nanoparticles, has the potential to improve the stability and targeting of drugs in the body. For example, the overproduction of ROS during immune cell activation is a key factor in tissue damage in myocarditis. The mitochondrial dysfunction induced by ICIs is closely related to the production of ROS. Therapies targeting ROS aim to mitigate oxidative stress at the mitochondrial level, a critical site of ROS overproduction during immune activation. Mitochondria-targeted antioxidants, such as MitoQ and N-acetylcysteine (NAC), have demonstrated potential in reducing ROS levels and limiting myocardial injury. Advances in nanoparticle-based delivery systems now enable precise subcellular targeting, leveraging mitochondrial membrane potential and specific surface markers to enhance drug accumulation and efficacy. These strategies improve therapeutic stability and minimize systemic toxicity, offering a promising avenue for addressing ICI-induced cardiovascular toxicity while preserving immune function. Early studies suggest that reducing ROS may reduce the inflammatory cascade associated with myocarditis while preserving T cell function to control cancer (220). The combination of reprogramming therapy with other treatments, including lipid-lowering drugs, anti-inflammatory therapies, and immunosuppressive therapies, may offer a more effective strategy for reducing cardiovascular complications associated with ICI.

In conclusion, while current research has initially highlighted the potential of immunometabolism regulation in ICI-related cardiovascular toxicity, its practical application still requires verification through further mechanism exploration and large-scale clinical trials in the future.
